# Frasier Syndrome: A Rare Cause of Refractory Steroid-Resistant Nephrotic Syndrome

**DOI:** 10.3390/children8080617

**Published:** 2021-07-21

**Authors:** Yung-Chieh Huang, Ming-Chin Tsai, Chi-Ren Tsai, Lin-Shien Fu

**Affiliations:** 1 Department of Pediatrics, Taichung Veterans General Hospital, 1650 Taiwan Boulevard Sect. 4, Taichung 40705, Taiwan; huang1985john@yahoo.com.tw (Y.-C.H.); karentsai78@yahoo.com.tw (M.-C.T.); n20248@mail.vghtc.gov.tw (C.-R.T.); 2 Institute of Molecular Biology, National Chung Hsing University, Taichung 40227, Taiwan; 3 Department of Pediatrics, National Yang-Ming University, Taipei 11221, Taiwan

**Keywords:** Frasier syndrome, pediatrics, steroid-resistant nephrotic syndrome, *WT1*

## Abstract

Frasier syndrome is a rare disease that affects the kidneys and genitalia. Patients who have Frasier syndrome develop nephrotic syndrome (NS) featuring focal segmental glomerulosclerosis (FSGS) that is resistant to steroid treatment in early childhood. Male patients can have female external genitalia (pseudo-hermaphroditism) at birth and develop gonado-blastoma in their adolescence. Frasier syndrome is caused by mutations in the splice donor site at intron 9 of the Wilms’ tumor *WT1* gene; these mutations result in an imbalanced ratio of WT1 protein isoforms and affect the development of the urogenital tract, podocyte function, and tumor suppression. Here, we report on a patient with long-term refractory NS who developed a malignant mixed germ cell tumor arising in a gonado-blastoma of the ovary 8 years after the onset of proteinuria.

## 1. Introduction

Frasier syndrome is a rare disease caused by the mutation of the Wilms’ tumor (*WT1*) gene [1]. The *WT1* gene and its encoding protein play multiple essential roles in the embryonic development of multiple systems, including the urogenital system, central nervous system, and mesothelial organs [2]. *WT1* is expressed in certain cell types such as glomerular podocytes after birth. It is also a tumor suppressor gene. *WT1* gene mutations are related to several different syndromes, including WAGR (Wilms’ tumor, aniridia, genitourinary malformations, and mental retardation) syndrome with deletion at 11p13 [3], Denys–Drash syndrome with point mutation in the eighth or ninth exon [4], and Frasier syndrome with mutation in intron 9.

The *WT1* gene has 36 isoforms (in mammals), as determined using alternative splicing, RNA editing, and the use of alternative translation initiation sites [5], but the functions of most isoforms remain unclear. The mutation of the splice donor site within intron 9 can alter the alternative splicing of the KTS (lysine, threonine, serine) motif and change the ratio between two isoforms, that is, the WT1(+KTS) and WT1(−KTS) proteins [2,5]. The *WT1* mutated allele is dominant and only produces −KTS isoforms, which leads to a reduced +KTS/−KTS isoform ratio [5]. Patients with this type of mutation, which is also known as Frasier syndrome, can present symptoms including steroid resistance nephrotic syndrome (SRNS) with FSGS, pseudo-hermaphroditism, and gonado-blastoma development in the second decade of life. To date, no study has reported on Frasier syndrome cases in Taiwan and fewer than 150 cases of Frasier syndrome have been reported worldwide [6].

## 2. Case Report

A 6-year-old girl presented to our pediatric nephrology outpatient clinic with proteinuria that was discovered during an elementary school health screening. No noteworthy abnormal findings, including any pertaining to her exo-genitalia, were observed during her physical examination. Her spot urine protein/creatinine ratio (UPCR) was 1.34 g/g with a 24-h urine protein of 0.66 g. Her renal sonography revealed increased echogenicity in both of her kidneys. Two months later, her proteinuria was still present with a UPCR of 2.1 g/g. At this point, we suggested a further survey, specifically a renal biopsy. The patient and her parents sought a second opinion at another medical center where the patient underwent a renal biopsy, which revealed focal segmental glomerulosclerosis (FSGS), with two sclerosis glomeruli out of seven. The second medical center arranged several sessions of methylprednisolone (MTP) pulse therapy for her nephrotic syndrome (NS) followed by daily oral steroid administration; however, her heavy proteinuria persisted. She received another renal biopsy 2 years later, and the pathology still showed FSGS (4/67 glomeruli). In the next 2 years, multiple regimens were attempted, including MTP pulse therapy, oral prednisolone, mycophenolate mofetil, tacrolimus, and abatacept, but the patient’s proteinuria progressed gradually with her UPCR increasing to 4.01 g/g. The patient’s regular medical treatment was halted when she reached 12 years old, and she started receiving alternative herbal therapy.

**Figure 1:**
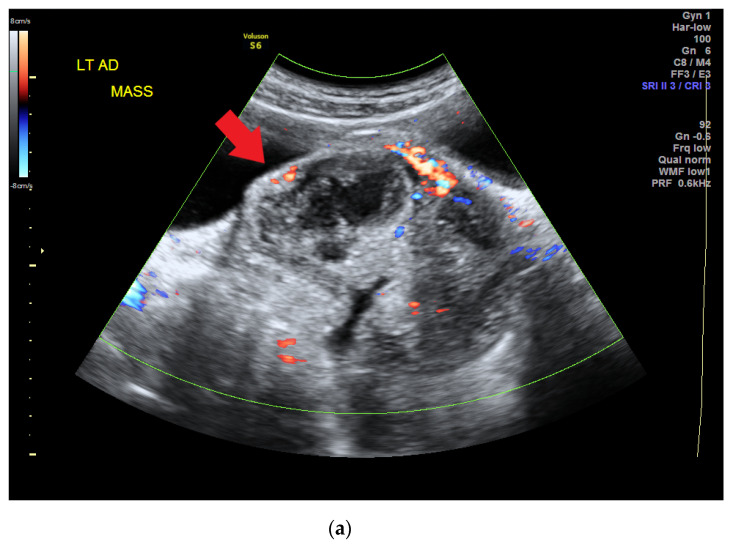

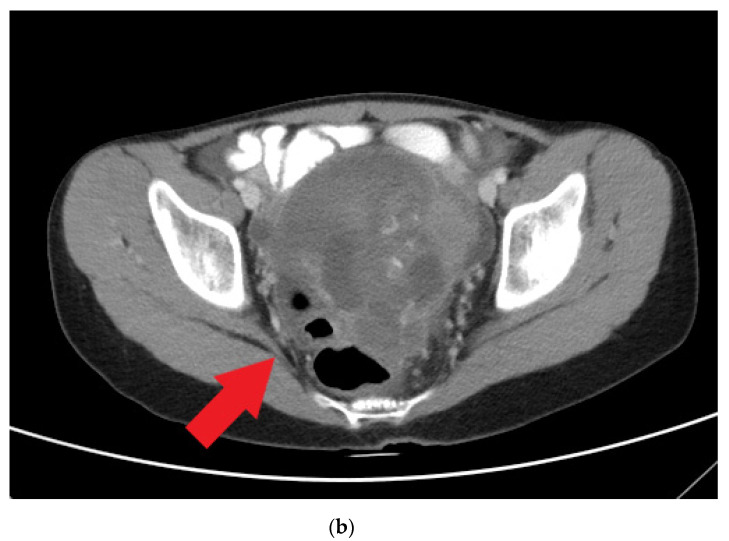
(**a**) Heterogeneous adnexal mass confirmed by sonography (red arrow). (**b**) Computed tomography scan revealing a lobulated mass lesion (maximum diameter of 10.1 cm) with cystic and solid components in the pelvis (red arrow).

The patient visited a gynecology clinic after experiencing a prolonged menstrual period of up to 1 month when she was 14 years old (she had her menarche at 12 years old). Preliminary sonography conducted at the clinic revealed a left ovarian tumor, and she was referred to our hospital. A heterogeneous adnexal mass was confirmed by sonography (Figure 1a) at our gynecology outpatient clinic. A computed tomography (CT) scan revealed a lobulated mass lesion (maximum diameter: 10.1 cm) with cystic and solid components in her pelvis (Figure 1b). Her serum tumor marker results were as follows: CA-125, 77.96 unit/mL (normal range: <35.0 unit/mL); CA-199, 57.01 unit/mL (normal range: <34.0 unit/mL); alpha fetal protein, 94.88 ng/mL (normal range: <7 ng/mL). At this time point, her serum albumin was 2.2 g/dL, cholesterol 183 mg/dL, triglyceride 133 mg/dL.

**Figure 2:**
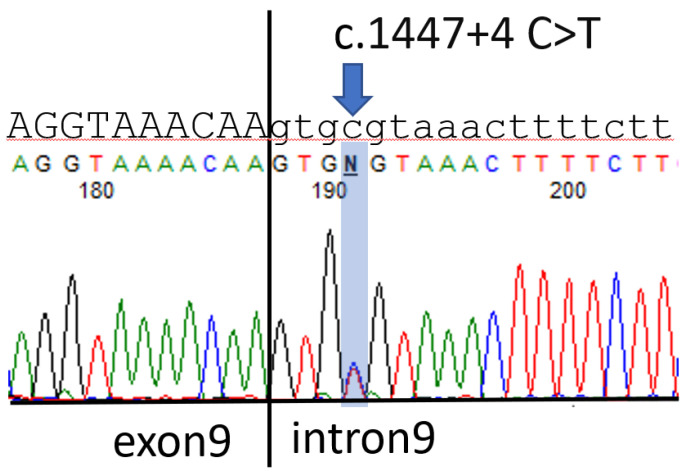
The uppercase and lowercase letters represent the sequences of exon9 and intron9 of WT1 gene respectively. Arrow indicates the c.1447 + 4 C > T mutation.

The patient received cytoreductive left salpingo-oophorectomy 1 week after the CT scan. The pathology report revealed a malignant mixed germ cell tumor arising in a gonado-blastoma of the ovary; the tumor was composed of 75% dysgerminoma, 10% immature teratoma, 10% embryonal carcinoma, and 5% yolk sac tumor. A further survey revealed that the patient had chromosome karyotype 46, XY, with the *SRY* gene being present in polymerase chain reaction (PCR) tests; however, the patient was observed to have grossly normal female external genitalia. Suspecting that the patient had Frasier syndrome, we conducted a genetic analysis that revealed a point mutation in intron 9 of the *WT1* gene, c.1447 + 4 C > T (Figure 2).

The patient received chemotherapy over the next 3 months with regimens (bleomycin, etoposide, cisplatin) implemented per the National Comprehensive Cancer Network (NCCN) guidelines for malignant germ cell tumor. Right salpingo-oophorectomy was performed 1 month after she completed her chemotherapy, and gonado-blastoma was observed in a pathology test. At this time point, her serum albumin was 3.7 g/dL, cholesterol 202 mg/dL, triglyceride 93 mg/dL. To date, she has been attending follow-up for 3 years and no signs of disease recurrence have been observed. We prescribed regular ezetimibe/atorvastatin for her hyperlipidemia and angiotensin-converting enzyme inhibitors (ACE-Is) for her proteinuria, though the patient did not have hypertension according to her age. No immunosuppressants were used. The patient still had heavy proteinuria (UPCR of up to 11.7 g/g) but no notable gross edema. Daily albumin replacement was prescribed for 3 days after her surgery, and none since then. Her serum albumin level has remained between 2.7–3.7 g/dL to this date. Her serum creatinine level was between 0.64–0.83 mg/dL in the recent 3 years.

The presentations of this patient are summarized in Table 1.

## 3. Discussion

No standard treatment currently exists for Frasier syndrome, either in the nephrology or oncology aspects. The use of cyclosporine is proposed due to its antiproteinuric effect in stabilizing the actin cytoskeleton of kidney podocytes by blocking the calcineurin-mediated dephosphorylation of synaptopodin [7]. Remission of proteinuria with the use of cyclosporine was reported in some cases [8,9]. However, the increased risk of malignancy should also be considered [8]. We did not administer cyclosporine to our patient because her renal function remained intact. Instead, we used ACE-Is to alleviate her proteinuria. ACE-Is and angiotensin receptor blockers (ARBs) have been proven to be effective in treating pediatric SRNS [10]; Chiba et al. [9] reported partial remission and normal renal function in a patient who had Frasier syndrome and underwent a combined treatment that included low-dose cyclosporine, ARB, and ACE-I.

Early elective bilateral gonadectomy is recommended in patients with Frasier syndrome [11]. The literature has reported patients to be tumor-free in long term follow-up after surgery and appropriate chemotherapy [8,9,12], but dysgerminoma recurrence was report by Mestrallet et al. [13]. Regular tumor marker, abdominal sonography or CT scan should be routinely evaluated.

The renal function of patients who have Frasier syndrome vary but usually declines gradually. If interventions are not performed, Frasier syndrome can lead to end-stage renal disease (ESRD) that requires dialysis in late childhood or adolescence, although this can occur as early as 4 years old [14]. In the cases where ESRD was reported, kidney transplantation was performed [14,15]. Preemptive kidney transplantation has been reported in some cases [12,13]. However, the opportunity of preemptive kidney transplantation is sparse without a living donor from family members [16]. The weight of benefits and risks should be well informed and discussed with the patient and potential relative donors; genetic counseling may provide more information to help make joint decisions [17]. Daily immunosuppressants are required for patients who received kidney transplantation to prevent rejection. Opportunistic infection, post-transplant lymphoproliferative disorder and cancer recurrence are major complications with immunosuppressants [18]. Considering the underlying malignancy potential in patients with Frasier syndrome, sufficient transplantation waiting time is essential after cancer treatment with surgery and chemotherapy [13]. In our patient, preemptive kidney transplantation is not taken into consideration since her renal function remained intact.

The diagnosis of NS is based on clinical presentations including heavy proteinuria, hypoalbuminemia, edema, and hyperlipidemia. Childhood NS is usually caused by minimal change disease and responds well to corticosteroids [19]. SRNS is defined as NS whereby remission is not induced within 4 weeks of daily corticosteroid therapy [20], and the most common cause of SRNS is FSGS. At least 33 genes are known to be associated with SRNS [21]; routine genetic tests are recommended if a patient is diagnosed with NS at less than one year old, but such tests are not recommended for patients in the classic age range (1–8 years old) with uncomplicated presentations [22]. Several algorithms are proposed for genetic testing with respect to SRNS [22]. The candidate genes include *NPHS1*, *NPHS2*, *WT1*, and *LAMB2* in infants [23]; and *NPHS2*, *WT1*, *TRPC6*, *ACTN4*, and *INF2* in adolescents [24]. However, test availability, cost, and the prevalence of varying candidate genes should be considered in clinical practice. For our patient, genetic analysis was not performed until she presented with extrarenal manifestation. Alternatively, G-banding karyotyping and *SRY* gene screening cost less and are accessible in most clinical settings; moreover, they can be detected in antenatal examinations [5]. Aucella et al. conducted a genetic survey of 32 female patients who had SRNS and were younger than 18 years old, and they discovered that two girls presented a 46 XY karyotype with streak gonads [25]. *WT1* splice mutations may not be rare in females under 18 years of age who have SRNS; prior to genetic testing, G-banding karyotyping and *SRY* gene screening may be considered for the differential diagnosis of SRNS.

In conclusion, we present a patient who had SRNS in childhood and gonado-blastoma in adolescence and was subsequently diagnosed with Frasier syndrome with genetic mutation in intron 9 of the *WT1* gene, c.1447 + 4 C > T. We believe that this is the first case of Frasier syndrome to be reported in Taiwan, although Frasier syndrome may be underdiagnosed here due to its clinical rarity. The differential diagnosis of SRNS remains a challenge, and Frasier syndrome ought to be considered as a cause.

## Figures and Tables

**Table 1:** Brief summary of this patient’s presentations.

Time (The Patient’s Age)	Symptoms	Laboratory Findings	Image Finding	Pathological Findings	Management
6 years old	Proteinuria during an elementary school health screening	UPCR: 1.34 g/g 24-h urine protein: 0.66 g	Renal sonography: increased echogenicity bilaterally		
2 months later		UPCR: 2.14 g/g		Renal biopsy: FSGS 2/7 glomeruli involved. IF *: negative EM **: 70% podocyte effacement.	MTP pulse therapy
8 years old	Persistent proteinuria			Renal biopsy: FSGS 4/67 glomeruli involved. IF *:negative EM **:70% podocyte effacement.	Tried MTP pulse therapy, oral prednisolone, mycophenolate mofetil, tacrolimus, and Abatacept; her proteinuria persisted
12 years old		UPCR: 4.01 g/g			regular medical treatment was halted
14 years old	Prolonged menstrual period of up to 1 month	CA-125, 77.96 unit/mL; CA-199, 57.01 unit/mL; alpha fetal protein, 94.88 ng/mL Serum albumin: 2.2 g/dL, cholesterol: 183 mg/dL, triglyceride: 133 mg/dL	CT scan: a lobulated mass lesion, maximum diameter: 10.1 cm	Malignant mixed germ cell tumor: 75% dysgerminoma, 10% immature teratoma, 10% embryonal carcinoma, and 5% yolk sac tumor	cytoreductive left salpingo-oophorectomy
Following 3 months					Chemotherapy Regular ezetimibe/atorvastatin and ACE-I
1 month later		Genetic test: point mutation in intron 9 of the WT1 gene, c.1447 + 4 C > T; serum albumin: 3.7 g/dL, cholesterol: 202 mg/dL, triglyceride: 93 mg/dL		Gonadoblastoma (right side)	Right salpingo-oophorectomy
To this date		UPCR: 11.7 g/g; Serum albumin between 2.7–3.7 g/dL			Regular ezetimibe/atorvastatin and ACE-I

* IF: immunofluorescence study. ** EM: electron microscope.

## Data Availability

Not applicable.
